# Both-Bone Forearm Fractures in Children with Minimum Four Years of Growth Remaining: Can Cast Achieve a Good Outcome at Skeletal Maturity?

**DOI:** 10.5704/MOJ.1711.009

**Published:** 2017-11

**Authors:** D Hadizie, I Munajat

**Affiliations:** Department of Orthopaedics, Universiti Sains Malaysia, Kubang Kerian, Malaysia

**Keywords:** forearm fracture, both-bone, functional outcome, children, skeletal maturity

## Abstract

**Introduction:** Both-bone forearm fractures in children can be treated non-operatively with a cast. Most previous studies have shown favourable outcome; however, information on the functional outcome after skeletal maturity is still scanty. Therefore, this study was conducted to determine the functional outcome after skeletal maturity in fractures with at least four years of growth remaining.

**Materials and Methods:** This retrospective study was conducted from March 2012 until March 2013. Age at the time of fracture was taken as until 10 years for females and until 12 years old for males with at least four years of growth remaining. Fractures occurring in the diaphysis were included in the study. Functional outcomes were assessed at or after skeletal maturity.

**Results:** Forty-four children fulfilled the criteria. The ages of the youngest and the oldest at the time of fracture was five and 12 years old respectively. Follow-up of the male and female patients were 7.4 years and 5.5 years respectively. There was a significant difference between post-reduction angulation and angulation at skeletal maturity of the radius and ulna (p<0.001). Out of 44 patients, 39 had excellent and five had good functional outcomes. No patient had fair or poor functional outcome. There was no association between the functional outcome and the angulation of forearm bones after skeletal maturity. Age at the time of fracture had a significant association with the functional outcome.

**Conclusion:** Non-operative treatment of both-bone diaphyseal forearm fractures in a cast has good to excellent functional outcomes in children who still have four years of growth remaining.

## Introduction

Forearm fractures in children can be treated with closed reduction and immobilisation since these fractures have good remodelling capability for correcting the angular deformity^1,2^. As much as 3% of all paediatric fractures are attributed to fractures involving the diaphysis of the radius and ulna^3^. Successful outcomes are based mainly on the restoration of pronation and supination. Most previous studies on forearm fractures in children showed favourable outcome during follow-up. However, the information on outcome measured after skeletal maturity is still scanty.

The acceptable degree of angulation at initial reduction at different segments of the forearm bones is still an issue. The remodelling capability is known to be better for younger children. As the child grows, this advantage may diminish, and the remodelling potential may not be sufficient to correct the deformity fully before skeletal maturity. The question now is how much time before skeletal maturity is considered enough for the angulation to be satisfactorily corrected by remodelling with a favourable functional outcome.

There is also some controversy regarding the functional outcome of forearm fractures in children. The unsatisfactory functional outcome documented before the skeletal maturity might not be accurate since the bone has not stopped remodelling. We believe that the angulation may not need to be fully corrected to obtain good or excellent functional outcome. We also believe that children with some residual angulation may still be able to have a good outcome at skeletal maturity.

Therefore, this study was conducted to determine the functional outcome specifically at skeletal maturity of both-bone forearm fractures in children treated non-operatively. We also wanted to determine whether the minimum four years’ duration allocated before the skeletal maturity was adequate for the bone to remodel and yield good functional outcome at skeletal maturity. The other objective was to study the factors that might be significantly associated with the functional outcome at skeletal maturity.

## Materials and Methods

This retrospective study was conducted in a single institution at our centre from 25th March 2012 until 24th March 2013, looking into children with both-bones forearm fracture previously treated non-operatively with a cast. The study was approved by the Research Ethics Board of Medical Sciences at our centre. The inclusion criteria were fractures of both forearm bones, a diaphyseal fracture which defined as a fracture occurring within the middle 3/5th of the forearm, complete fracture and fractures treated non-surgically using full-length cast. Age at the time of fracture was taken as until ten years for a female and until 12 years old for a male with at least four years of growth remaining. Exclusion criteria were a metaphyseal fracture, single bone fracture, incomplete fracture, Galeazzi fracture, Monteggia fracture, previous operative intervention to the fractures and refracture.

Cases were identified from the patients’ medical file and radiology records. List of forearm radiographs with both-bone fractures available in the PACS-IW system (computerised and centralised radiological program in HUSM) was carefully evaluated. Patients who fulfilled the radiological inclusion and exclusion criteria were selected. Other information regarding the patients’ data and treatment details were obtained from the patients’ medical files. Age of assessment of functional outcomes was taken at or after skeletal maturity, which was at or more than 14 years old for female and at or more than 16 years old for a male. The selected patients were called and their willingness and consent taken to be involved in this study.

**Fig. 1:**
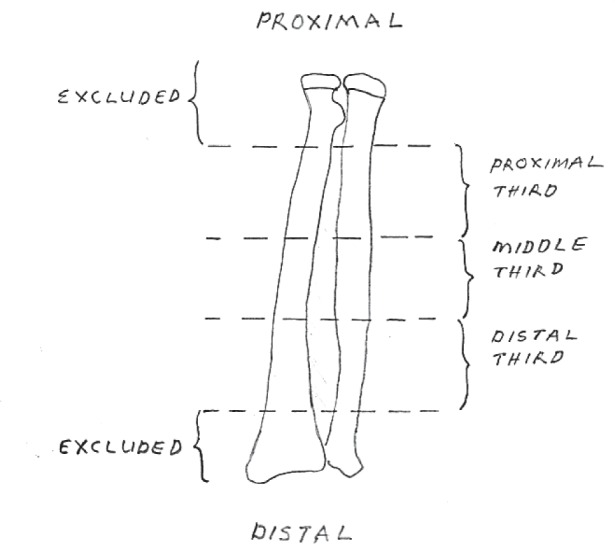
An illustration showing the division of forearm into 5 regions and only the fractures occurring within central 3/5 were included in the study.

Location of the fracture, based on the radiograph, was divided into proximal third, middle third and distal third. Fractures occurring in the diaphyseal region, which was within central 3/5th of radius and ulna, were included in the study (Fig. 1). The patients were assessed based on the radiological and functional outcomes.

**Fig. 2:**
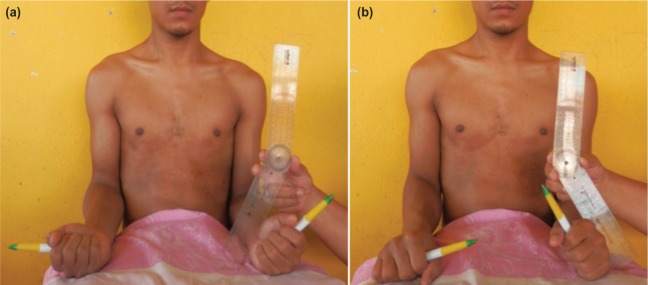
Photos showing the assessment of the degree of supination (a) and pronation (b).

Supination and pronation of the affected forearm were measured using hand-held protractor goniometer with two moveable arms of 20 centimetres. The unaffected forearm was used as a normal reference. Before each measurement, the patients were required to sit in an upright position. The elbow was positioned firmly against the torso to eliminate compensating forearm rotation using movements of the elbow and shoulder. The elbow was flexed to 90 degrees with the forearm in mid-position and the wrist in neutral while the hands were holding the pens in an upright position to help in better visualisation of both pronation and supination of the forearm (Fig. 2). For the measurement, one arm of the goniometer was lined up parallel to the upper arm of the patient, and the other arm of the goniometer was placed parallel to the distal third of the forearm. The ranges of pronation and supination of the affected forearm were measured in comparison with the unaffected forearm. The differences in the range of pronation and supination between affected and unaffected forearm were taken as the measurement.

The patients were assessed regarding the functional limitation with physical activity or activity of daily living according to the Price functional outcome grading which was either excellent, good, fair or poor. Excellent was defined as no complaint with strenuous physical activity, for example sports activity, and/or loss of 10 degrees or less of forearm rotation. Good outcome was considered when mild complaint with strenuous physical activity, for example using a screwdriver and/or loss of 11-30 degree of forearm rotation. The fair outcome is when the patients had a mild subjective complaint during usual physical activity, for example opening the lid of jars or door and/or loss of 31-90 degree of forearm rotation and poor outcome was defined as non-fulfilment of all other results.

Anteroposterior and lateral radiographs on the affected forearm were taken according to the proper positioning and exposure suggested by Martensen *et al*^4^. The evaluation of the radiograph was made by measuring the post-reduction angulation and final angulation at skeletal maturity of both radius and ulna. The angulation was defined as the maximal angulation of each bone present on either the AP or lateral view^5,6^. The measurement was performed by using the measurement tools in the PACS-IW system (computerised and centralised radiological program).

To determine the reliability of the measurement we took into account the natural anatomical bowing present at the middle portion of the radius. From the study by Bowman *et al*^7^, the natural bowing of the radius showed an apex radial bow of 1.5 degrees in the proximal third, 6.0 degrees in the middle third and 1.7 degrees in distal third fractures. Based on that measurement, we applied a correction factor of 6 degrees apex radial to the anteroposterior measurement of the middle third of radius (apex radial measurements decreased by 6 degrees, and apex ulnar measurements were increased by 6 degrees). Based on the result of that study, we did not apply the correction for the distal and proximal radius measurement because their mean values were within the accepted margin of reader error of ±5 degrees.

**Fig. 3:**
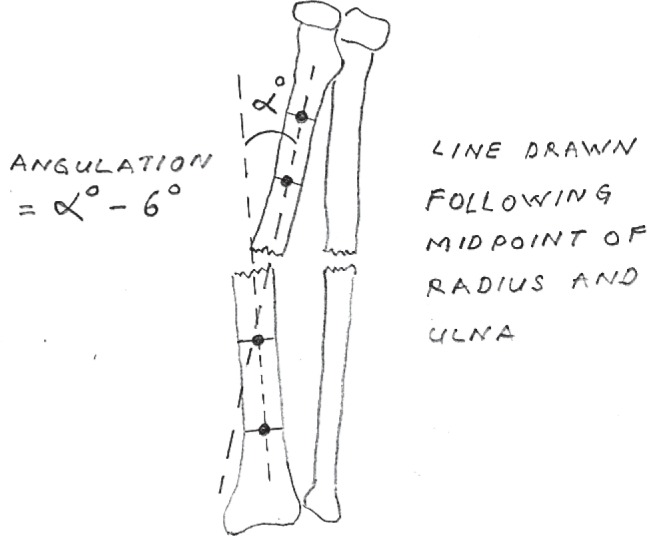
An illustration showing the measurement of fracture angulation.

The degree of angulation was measured by drawing a perpendicular line following two midpoints of the radius and ulna bone for each segment of the fracture. The angle (in degrees) that formed in between those perpendicular lines from each segment of the fractures was taken as the reading. The same procedure was performed for both anteroposterior and lateral radiograph of radius and ulna, and the higher measurement for each bone was taken as the final degree of angulation. For example, if the angulation of the radius in the anteroposterior view was 10 degrees and in the lateral view was 15 degrees, the 15 degrees was taken as the final angulation of the radius. Furthermore, in the middle third of radius with the apex of the fracture was towards the ulna, 6 degrees would be added to the measurement, and if the apex of the fractures was towards the opposite direction, 6 degrees would be deducted from the measurement (Fig. 3). Each of the measurement was made twice by the same examiner, and the mean was taken as final.

All statistical analyses were performed using the Statistical Program for Social Sciences SPSS version 20. For descriptive analysis, numerical variables were described as a mean and standard deviation and p-Value obtained from independent T-test. For univariate analysis, simple logistic regression was used to determine the potential associated factors for outcome. Any factors with p-Value less than 0.05 was considered significant.

## Results

Among children presented with forearm fractures between 1992 and 1999, 44 fulfilled the criteria for this study. Individual consent was obtained from all patients and parents. There were 36 males (81.8%) and eight females (18.2%). The youngest age at the time of fracture was five years old and 12 years old was the oldest. All patients had at least four years of growth remaining before achieving skeletal maturity. Mean follow-up of the male and female patients were 7.4 years (ranges 4-11 years) and 5.5 years (ranges 4-9 years) respectively. The majority of the patients sustained the injury at the age of six years. Both sides of the forearms were almost equally involved. In this study, only one patient (2.3%) had sustained an injury at proximal third of forearm, 20 patients (45.5%) at middle third and 23 patients at distal third of forearm (52.2%).

The mean radius angulation after reduction was 10.3 degrees (ranges 3 to 24 degrees) while at skeletal maturity, the angulation was corrected to within the range of 0 to 11 degrees with the mean of 2.8 degrees (Table I). For the ulna, post reduction angulation was within 1 to 19 degrees (mean of 8.9 degrees) with improvement to within 0 to 8 degrees (mean of 2.3 degrees) at skeletal maturity. There was a significant difference between post-reduction angulation and angulation at skeletal maturity of the radius and ulna (p<0.001) (Table II). The mean angular corrections for radius and ulna were 7.4 degrees (72% correction) and 6.7 degrees (75% correction) respectively.

**Table I.** Summary of the results of radiographic changes of the radius and ulna and the functional outcome at skeletal maturity (n=44)

Case no	Age and gender at time of fracture (years/sex)	Radius-post reduction (degree)	Radius-at skeletal maturity (degree)	Ulna-post reduction (degree)	Ulna-at skeletal maturity (degree)	Forearm ROM limitation (supination, pronation) (degree)	Price grading (Functional outcome at skeletal maturity)
1	7/M	12..0	2.0	6.0	0.0	0.0,0.0	Excellent
2	6/M	15.0	2.0	1.0	0.0	0.0,0.0	Excellent
3	10/M	24.0	10.0	13.0	4.0	6.0,4.0	Excellent
4	7/M	21.0	3.0	10.0	1.0	0.0,0.0	Excellent
5	10/F	10.0	3.0	14.0	4.0	4.0,0.0	Excellent
6	9/M	8.0	0.0	4.0	0.0	0.0,0.0	Excellent
7	11/M	9.0	3.0	13.0	5.0	4.0,6.0	Excellent
8	9/M	9.0	1.0	11.0	2.0	0.0,0.0	Excellent
9	9/F	11.0	11.0	11.0	7.0	0.0,0.0	Excellent
10	7/M	10.0	1.0	19.0	3.0	0.0,0.0	Excellent
11	5/M	13.0	0.0	8.0	0.0	0.0,0.0	Excellent
12	7/M	7.0	6.0	12.0	4.0	0.0,4.0	Excellent
13	10/M	9.0	3.0	6.0	1.0	10.0,6.0	Excellent
14	7/M	12.0	2.0	7.0	0.0	0.0,0.0	Excellent
15	6/M	3.0	0.0	6.0	2.0	0.0,0.0	Excellent
16	6/F	13.0	4.0	9.0	2.0	0.0,0.0	Excellent
17	5/M	13.0	2.0	10.0	3.0	0.0,0.0	Excellent
18	10/M	9.0	0.0	10.0	0.0	10.0,10.0	Excellent
19	11/M	8.0	0.0	5.0	0.0	6.0,4.0	Excellent
20	9/M	12.0	4.0	10.0	3.0	0.0,0.0	Excellent
21	8/M	7.0	0.0	5.0	0.0	0.0,0.0	Excellent
22	10/M	14.0	5.0	8.0	0.0	4.0,4.0	Excellent
23	10/M	3.0	0.0	15.0	0.0	10.0,20.0	Good
24	11/M	8.0	1.0	11.0	3.0	6.0,10.0	Excellent
25	8/M	11.0	4.0	11.0	3.0	0.0,0.0	Excellent
26	8/F	8.0	2.0	5.0	1.0	0.0,0.0	Excellent
27	8/M	4.0	0.0	11.0	5.0	0.0,0.0	Excellent
28	10/M	14.0	5.0	9.0	2.0	6.0,4.0	Excellent
29	9/F	7.0	2.0	8.0	2.0	4.0,0.0	Excellent
30	9/M	5.0	0.0	5.0	0.0	0.0,0.0	Excellent
31	7/F	5.0	0.0	6.0	0.0	0.0,0.0	Excellent
32	11/M	10.0	3.0	12.0	2.0	6.0,10.0	Good
33	9/M	10.0	3.0	3.0	0.0	0.0,0.0	Excellent
34	12/M	8.0	4.0	9.0	2.0	10.0,6.0	Excellent
35	11/M	8.0	5.0	12.0	2.0	10.0,6.0	Excellent
36	9/M	10.0	4.0	5.0	0.0	0.0,0.0	Excellent
37	11/M	13.0	5.0	15.0	8.0	10.0,10.0	Good
38	8/M	20.0	7.0	9.0	6.0	0.0,0.0	Excellent
39	8/F	14.0	5.0	9.0	2.0	0.0,0.0	Excellent
40	11/M	6.0	2.0	6.0	3.0	14.0,10.0	Good
41	9/M	11.0	4.0	9.0	1.0	0.0,0.0	Excellent
42	10/M	7.0	2.0	6.0	2.0	6.0,10.0	Good
43	10/F	10.0	9.0	11.0	5.0	6.0,0.0	Excellent
44	9/M	11.0	0.0	8.0	5.0	0.0,0.0	Excellent
Mean	8.8	10.3	2.8	8.9	2.3	3.0 (0.0-14.0),	
	(5-12)	(3.0-24.0)	(0.0-11.0)	(1.0-19.0)	(0.0-8.0)	2.8 (0.0-20.0)	

* The study respondents’ characteristics according to the functional outcome grading by Price; Excellent: no complaint with strenuous physical activity and/or loss of 10 degrees or less of forearm rotation, Good: mild complaint with strenuous physical activity and/or loss of 11-30 degree of forearm rotation; Fair: mild subjective complaint during usual physical activity and/or loss of 31-90 degree of forearm rotation, Poor: all other result.

**Table II:** Angulation of the radius and the ulna at post reduction and at skeletal maturity (n=44)

Parameter	Mean of post reduction angulation (SD)	Mean angulation at skeletal maturity (SD)	Mean differences (95% CI)	t-stat	p-Value
Radius	10.3 (4.3)	2.8 (2.6)	7.4	14.0	<0.001
			(6.4,8.5)	(43)	
Ulna	8.9 (2.6)	2.3 (2.1)	6.7	16.5	<0.001
			(5.8,7.5)	(43)	

* Paired sample t-test

The limitation in supination of the forearm at skeletal maturity was from 0 to 14 degrees with the mean of 3 degrees. For pronation, the limitation was in the range of 0 to 20 degrees with the mean of 2.8 degrees.

Out of 44 patients, 39 had excellent functional outcome, and five had good result according to functional outcome grading by Price (Table III). No patient had fair or poor functional outcome. All 44 patients with excellent results had lost 10 degrees or less of forearm rotation. In five patients with good results, two had lost 11-30 degrees of forearm rotation while three had lost 10 degrees or less but grouped under good rather than excellent outcome since patients had mild complaints of pain and fatigue with strenuous activities.

**Table III:** The functional outcome of the forearm according to Price grading at skeletal maturity (n=44)

Parameter	n (%)
Excellent	39 (88.6)
Good	5 (11.4)
Total	44

**Fig. 4:**
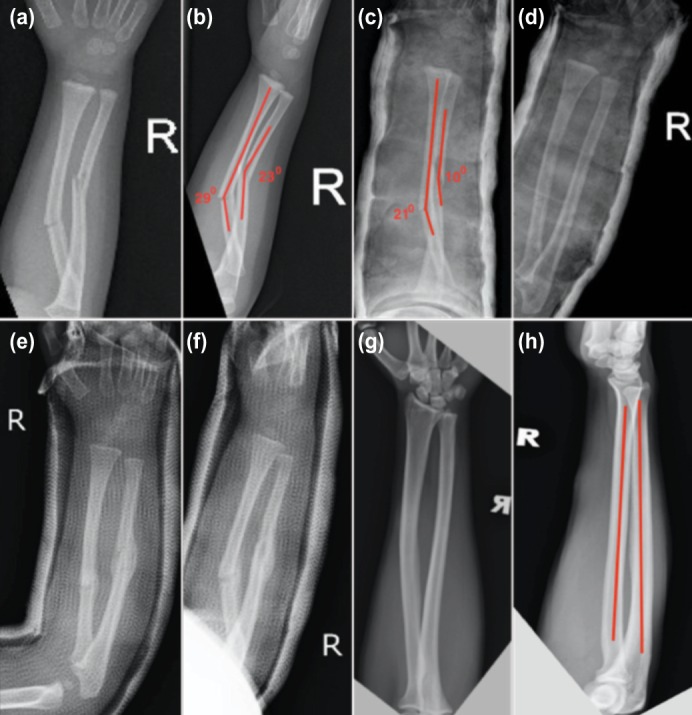
Photos showing the pre-reduction AP and lateral radiographs (a and b) of Case 4 with 29 degrees maximum angulation of radius and 23 degrees maximum angulation of ulna on lateral view (b), reduction of the angulation to 21 degrees for radius and to 10 degrees for ulna on lateral view upon casting (c), relatively good bone alignment after closed reduction on AP view (d), remodeling and healing of the bones with time (e and f) and complete remodeling with correction of the fracture angulation at skeletal maturity (g and h).

One patient had 20 degrees’ limitation of forearm pronation. Two patients had 6 to 10 degrees of supination loss and 4 to 10 degrees of pronation loss despite complete remodeling of the radius and ulna with no angulation. The fracture configurations pre- and post-reduction, the fracture healing and remodeling, and the bone realignment at skeletal maturity are illustrated in Figure 4 using Case 4 as a case illustration.

In simple logistic regression, there was no significant association in the angulation of radius and ulna post-reduction, the angulation of radius and ulna after skeletal maturity, and site of the fracture with the forearm rotation and the functional outcome (Table IV). However, age at time of fracture had significant association with the functional outcome (Table IV) (Simple logistic regression; crude odds ratio = 3.299; 95% CI; p-value = 0.034). From this model, children with a 1-year increment of age at the time of fracture will have 3.3 times the odds to have a good outcome in this study. The older the age of the child at the time of fracture the more likely the child was noted to have good rather than excellent functional outcome.

**Table IV:** Simple logistic regression of the associated factors for the functional outcome at skeletal maturity

Parameter	Crude OR (95% CI)	p-Value
Age at time of fracture	3.3 (1.1,9.9)	0.0
Pre ulna angulation	1.2 (0.9,1.5)	0.2
Pre radius angulation	0.8 (0.6,1.1)	0.2
Post ulna angulation	1.5 (1.0,2.3)	0.1
Post radius angulation	0.92 (0.6,1.4)	0.7
Location		
Proximal to distal	0.0 (0.0,__)	1.0
Middle to distal	0.7 (0.1,5.0)	0.8

## Discussion

Both-bone forearm fractures in younger children can still be managed nonoperatively despite the emergence of titanium elastic nails for surgical intervention. Their younger age and tremendous remodelling capability are the main advantages for them to heal successfully. Unless the fracture angulation has fully corrected, the outcome of the treatment ideally has to be assessed at or after skeletal maturity to ensure that the bone had undergone full remodelling before skeletal maturity. The assessment is particularly relevant when the fracture occurs near skeletal maturity with only a few more years remaining. Previous literatures assessing the outcome at skeletal maturity are still lacking. Therefore, in this study, we looked at the functional outcome specifically at skeletal maturity together with the residual angulation if any of the both bone forearm fractures in children treated nonoperatively.

The maximum age at the time of fracture was taken at 10 and 12 years old for girl and boys respectively. This was to ensure that all the selected patients had at least four years of remaining growth before they reached skeletal maturity, which was expected at 14 years old for a girl and 16 years old for a boy^8^. Other studies were assessing majority of their patients within two to three years of follow-up rather than after skeletal maturity^5-7,9,10^. In our study however, we were assessing all of our patients after they had reached skeletal maturity. We believed that by having at least four years of growth remaining, we were giving ample time for the bone to remodel. As long as the physis is still open, the remodelling process can take place, and the possibility of better outcome can be achieved after skeletal maturity^11^.

The angular deformity improved once the patients reached skeletal maturity and about 72 to 75% of correction was observed in our study. In comparison with other studies, 50% correction of angulation was possible for shaft fractures in children less than eight years of age with less than 20 degrees of angulation^12-14^. Our study showed a lesser degree of residual angulation than Naziri and Daruwalla *et al* studies^5,9^. In their studies, the final assessment was not conducted after the patients had reached skeletal maturity and their results showed a higher degree of angulation. Naziri *et al*^5^, showed that the final angulation in their series ranged from 0 to 16 degrees for the radius and 0 to 20 degrees for the ulna. The age range of their study populations was within 4 to 12 years old^5^.

Accepted guidelines for children with more than two years of growth remaining are 15 degrees of angulation^6,15^. There are remaining growth and remodelling in the children after the fracture union as long as the physis is still open. Furthermore, all our patients had at least four years of remaining growth from the time of fracture before they reached skeletal maturity.

In our series, the worst angulation for the radius after reduction was 24 degrees in a 10 years old child. At maturity, he still had 10 degrees of residual angulation. However, the functional result was excellent, and he had no complaint and no limitation on his strenuous or daily activities. Our worst post-reduction angulation of the ulna was 19 degrees in a 7 years old child with good remodelling leaving only 3 degrees of residual angulation at skeletal maturity. He achieved an excellent result as well. Price *et al*^10^, accepted up to 15 degrees of angulation for children less than eight years old and only ten degrees for the patients more than eight years old for distal and middle third of forearm fractures. Hughston *et al*^16^, showed in his series that 10 years old children with 30 to 40 degrees of angulation still had an excellent outcome. Zionst *et al*^6^, also showed that even with residual angulation the functional result was still satisfactory. Naziri *et al*^5^ also concluded in their study that in children less than 10 years old, angulation of up to 20 degrees was still acceptable. These studies have shown that the acceptable limit for reduction was still inconclusive. Based on our study, we concluded that up to 20 degrees of angulation in diaphyseal forearm fracture was still acceptable in children less than eight years old to achieve good to excellent functional outcome. It was also noted that the degree of deformity either post-reduction or at the skeletal maturity has no association with the functional outcome.

Age is the only factor proven to have a significant association with the functional outcome in our study. The younger age group seems to have a more favourable outcome, which is supported by Bowman *et al*^7^, in which they allow a larger degree of angulation as an acceptable reduction in a younger age patient. Price *et al*^17^, recommended eight years of age while Noonan *et al*^18^, recommended nine years old as their cut off point for decision making to accept a certain degree of angulation after closed reduction. Bowman *et al*^7^, allowed up to 20 degrees in female less than eight years old and male less than ten years old. However, only 10 degree of angulation was acceptable for female and male patients aged more than 8 and 10 years old respectively^7^. The younger children have a better outcome relatively because they still have more chance for bone remodelling after the fracture has united compared to those who sustained injuries at age closer to skeletal maturity.

Daruwalla *et al*^9^, reviewed 53 displaced forearm fractures in children with an average of three years follow-up and found that all the patients were asymptomatic and had no limitations in their activities even though 6% of them had lost more than 30 degrees of forearm rotation. This data was further supported by Hogstrom *et al*^14^, and Morrey *et al*^19^, who described that with the limitation of 60 degrees or less in the range of pronation and supination, patients seemed to be unaware of their incapacity due to good compensation by shoulder motion. Sinikumpu *et al*^20^, reviewed 47 nonoperatively treated both-bone forearm shaft fractures in children and found that the prono-supination of the forearm was not decreased in the long term, the grip strength was also equally as good as in the controls and the patients were satisfied with the outcome. In our study, the worst forearm rotation observed after skeletal maturity was 20 degrees far less than above studies. It might be the reason why we did not encounter any fair or poor result.

Proximal forearm fractures have a worse prognosis for the recovery of motion compared with midshaft or distal shaft fractures^9,10,21,22^. In our study, we only had one patient with proximal forearm fracture (2.3%). The rest were either midshaft or distal forearm fractures. Lack of proximal forearm fracture was the limitation in our study and was probably the reason why we could not statistically find any association between site of fracture and limitation of forearm motion. Since most were at middle and distal third, this might also have contributed to a better functional outcome in our study compared with other studies.

In our study, there were three patients with no angulation of radius and ulna at maturity, but they still had about six to ten degrees of supination loss and four to 20 degrees of pronation loss. Good bone remodelling with no angulation on radiograph may not correlate with the return of forearm rotation^10-13^. This poor correlation between angulation and functional outcome has been shown as well in few studies. In a study by Price *et al*^10^, a 13-year old girl with displaced forearm fracture and 10 degrees of radius residual angulation after nine years had a full range of forearm rotation. Another case who was also reported by Price *et al*^10^, revealed a 6-year old girl with severe fractures of both right radius and ulna had complete remodelling after four years follow up. However, she lost 30 degrees of forearm pronation despite having no residual angulation of radius and ulna^10^.

These findings have raised few theories regarding the factors that contribute to the limitation of forearm rotation even with complete remodelling and no residual angulation. Length discrepancies, encroachment of the interosseous space and displacement in the cases of closed treatment have been thought as the possible causes. Scarring of the surrounding soft tissue following the fracture produces some tension and encroachment in the interosseous membrane, and this will result in loss of a significant degree of forearm rotation^10^.

We did not consider the rotation of the fracture in our study based on the fact that it was difficult to measure rotational deformity from the radiograph accurately and the rotation was unlikely to be corrected by remodelling^9,23^. Furthermore, the rotational deformity was accepted within 0 to 45 degrees^11^. Creaseman *et al*^24^, did measure the rotational deformity of the fractures in his study but most other literature measured only the angulation of the fractures^6,7,9,24^. They had difficulty in assessing the rotational deformity in their study due to difficulty in getting true tuberosity view^24^.

## Conclusion

Non-operative treatment of both-bone diaphyseal forearm fracture with a cast, particularly in middle and distal third fractures, has good to excellent functional outcomes in children who still have four years of growth remaining. The degree of angulation post reduction and at skeletal maturity do not influence the functional outcome at skeletal maturity. Age at the time of fracture is the only factor proven to have a significant association with, and influence on, the functional outcome. A year’s increment of the age at the time of fracture will have 3.3 times the odds to have a less favourable outcome. The older the child at the time of fractures, the more likely is the child to have good rather than excellent functional outcome.
